# Depression in dementia with Lewy bodies: A comparison with Alzheimer's disease

**DOI:** 10.1371/journal.pone.0179399

**Published:** 2017-06-15

**Authors:** Pai-Yi Chiu, Chein-Wei Wang, Chun-Tang Tsai, Shin-Hua Li, Chih-Li Lin, Te-Jen Lai

**Affiliations:** 1 Department of Neurology, Show Chwan Memorial Hospital, Changhua, Taiwan; 2 Department of Neurology, Taichung Lin-Shin Hospital, Taichung, Taiwan; 3 Department of Guidance and Counseling, National Changhua University of Education, Changhua, Taiwan; 4 Institute of Medicine, Chung Shan Medical University, Taichung, Taiwan; 5 Department of Psychiatry, Chung Shan Medical University Hospital, Taichung, Taiwan; Chiba Daigaku, JAPAN

## Abstract

**Background:**

Depression is highly associated with dementia, and this study will compare the frequencies, severity, and symptoms of depression between dementia with Lewy bodies (DLB) and Alzheimer’s disease (AD).

**Methods:**

Frequency of depression was determined according to the DSM-IV criteria for major depression or the National Institute of Mental Health criteria for depression in AD (NIMH-dAD). Severity of depression were assessed using the Hamilton Depression Rating Scale, the Cornell Scale for Depression in Dementia, and the depression subscale in Neuropsychiatric Inventory. The rates of depressive symptoms were compared between AD and DLB.

**Results:**

A total of 312 patients were investigated (AD/DLB = 241/71). The frequency of major depression was significantly higher (p = 0.017) in DLB (19.7%) than in AD (8.7%). The higher frequency of depression in DLB was not reproduced by using the NIMH-dAD criteria (DLB: AD = 43.7%: 33.2%; p = 0.105). The severity of depression was higher in DLB than in AD according to the Hamilton Depression Rating Scale (p < 0.001) and the Cornell Scale for Depression in Dementia (p < 0.001). Among depressive symptoms, pervasive anhedonia had the highest odds ratio in DLB compared with AD.

**Conclusion:**

This is the first study using the NIMH-dAD criteria to investigate the frequency of depression in DLB. Our study shows that co-morbid major depression is more frequent in DLB than in AD. Pervasive anhedonia had the greatest value for the differential diagnosis of depression between DLB and AD.

## Introduction

Clinical studies investigating the prevalence and severity of depression associated with dementia have found that depression or depressive symptoms are among the most common behavioral and psychological symptoms of Alzheimer’s disease (AD) and dementia with Lewy bodies (DLB) [1–3]. Depression is a nonmotor manifestation that has been frequently described in the preclinical or early phase of Parkinson’s disease (PD) as well as of DLB [4,5,6]. Two thirds of dementia patients with depression at baseline were still depressed at the follow-up, more so in DLB than in AD [7]. Unfortunately, depression has a great impact on the outcomes of dementia patients and worsens the quality of life of dementia patients and their caregivers [8,9].

Depression is classically explained by the monoamine hypothesis, which proposes that depression is related to a deficit of monoamines, particularly norepinephrine (NE), serotonin (5-HT), and dopamine (DA) [10]. Recent functional imaging studies have indicated that depressed mood is associated with brain areas that receive innervation from serotonergic projections from the midbrain raphe nucleus and from the noradrenergic projections from the locus ceruleus, as well as from dopaminergic projections from the ventral tegmental area [10] Besides the association of depressed mood with a deficit of monoamines, the mesolimbic DA pathway is a key regulator of interest/pleasure and dysfunctioning of the pathway may underlie the depression in PD [10,11].

Pathological studies examining the neural correlates of depression in primary dementia have indicated cell loss in the locus ceruleus and substantial nigra [12]. Loss of rostral raphe neurons may also contribute to depression in DLB [13]. However, the neural substrates underlying depression in DLB and AD may be different. The major neurochemical difference between AD and DLB is in the dopaminergic metabolism. A postmortem study of brains by Piggott et al. from patients with PD, DLB, or AD, and elderly controls showed reduction in the dopamine concentration in the putamen of DLB and PD patients, while there was no change in AD patients [14]. This study also found that in DLB and PD, there is also reduced binding to the dopamine uptake sites (presynaptic receptors) in the putamen (57%). There are no changes in the uptake sites in AD as compared with the control values. Besides a dopaminergic metabolism, the serotonergic system also differs between DLB and AD [15–18]. These studies revealed that marked reduction of serotonin levels have been reported in the striatum, neocortex and frontal cortex; however, DLB patients with major depression had relatively preserved 5HT transporter re-uptake sites, compared to those without.

One methodological concern in studies of depression associated with dementia is that different rates of depression in dementia may be due to not only different dementia severity but also different assessment tools for depression. The DSM criteria for major depression and/or minor depression are frequently used for the determination of depression in patients with DLB and other dementia. Most previous studies on depression and/or other psychiatric symptoms associated with DLB had small sample sizes and were retrospective case note reviews using different methods [19]. Some studies have demonstrated that depression is more frequent in DLB than in AD [19–21]. However, some studies did not show the difference [22–24]. In addition to the DSM criteria for major depression, a workgroup convened by the National Institute of Mental Health (NIMH) proposed standardized diagnostic criteria for depression in AD, known as the NIMH-dAD provisional criteria [25,26]. The symptom profile of the NIMH-dAD criteria is similar to that of the DSM-IV criteria for major depression; however, although it omits the decreased ability to think and concentrate, it includes irritability, social isolation, and loss of pleasure in response to social contact replacing loss of interest in DSM-IV. Using the NIMH-dAD criteria, rates of depression in AD have been reported to range from 22 to 54% in other studies [26–28]. However, as yet, there is no study using the NIMH-dAD criteria to evaluate the depression rate in DLB.

Using other depression rating scales, such as the Neuropsychiatric Inventory (NPI) [7], the Montgomery and Åsberg Depression Rating Scale (MADRS) [7], and the Geriatric Depression Scale (GDS) [29], DLB showed more severe depression compared with AD. Regarding the depressive symptoms in DLB compared with those of AD, few studies have directly compared the symptoms among DLB and AD patients. A comparative study on autopsy-confirmed DLB and AD revealed that the depressive symptom distribution for AD and DLB did not differ across all symptoms [19].

Because there is still controversy regarding the results of the prevalence of depression and depressive symptoms associated with DLB and AD, we have designed a relatively complete comparative study to clarify the issue. In this study, we made the following hypotheses: compared with AD, depression in DLB is higher in frequency as well as in severity; using the DSM-IV major or the NIMH-dAD provisional criteria, the frequency of depression in DLB patients is higher than in AD patients; using the Hamilton Depression Rating Scale (HDRS) [30], the Cornell Scale for Depression in Dementia (CSDD) [31], and the NPI depression subscale [32], the depression in DLB patients is more severe than that in AD. Finally, based on the pathophysiological as well as the neurochemical differences between DLB and AD, we hypothesized that the depressive symptoms must be different between DLB and AD.

## Material and methods

### Participants

The study was conducted in a 750-bed regional hospital in central Taiwan. From February 10, 2010 to February 9, 2012, a consecutive series of outpatients with AD or probable DLB were enrolled in this study. The AD patients were diagnosed according to the criteria for primary degenerative dementia and AD in the fourth edition of the Diagnostic and Statistic Manual of Mental Disorders (DSM-IV). The diagnosis for probable DLB was made according to the consensus criteria developed by the third report of the DLB consortium [6]. According to the criteria, probable DLB is clinically diagnosed with the necessity of at least two of the three core features (fluctuation, visual hallucinations, and parkinsonism) or one core feature plus at least one of the three suggestive features (REM sleep behavior disorder, severe neuroleptic sensitivity, and low dopamine transporter uptake in basal ganglia).

### Assessment tools

All patients received a structured interview of baseline demographic data and clinical history, laboratory studies, and cerebral CT or MRI to rule out other possible causes of dementia. Severity of dementia was established using the Clinical Dementia Rating (CDR) scale [33]. Cognitive functions were assessed using the Chinese version of the Cognitive Abilities Screening Instrument (CASI C-2.0) [34] and the Mini-Mental State Examination (MMSE) [35]. The Mayo Fluctuation Composite Score (MFCS) [36] was used to assess the fluctuation of consciousness and the motor score of the DLB. The Unified Parkinson’s Disease Rating Scale (UPDRS) was used to assess motor functions [37]. Cognitive tests of all patients were performed by a trained neuropsychologist (Tsai CT). Dementia and subtype of dementia were made by a consensus meeting composed of two neurologists (Chiu PY and Chen PK), one geriatric psychiatrist (Lai TJ), and one neuropsychologist (Tsai CT).

### Diagnosis of DLB

All patients and their main caregivers were interviewed by a neurologist for the assessment of core and suggestive features. Fluctuation was diagnosed when a clinical history of fluctuation in cognition and a MFCS > 2 were both present. Visual hallucinations (VHs) were diagnosed when a clinical history of recurrent complex VHs was present. Recurrent VHs were defined as more than one episode within one month. Complex VHs were defined as well-formed people, animal, or with not only single geographical object or color. Parkinsonism was diagnosed when at least 2 of the following were present: bradykinesia, tremor, rigidity and postural instability. RBD was diagnosed when the minimal criteria for REM sleep behavior disorder in the International Classification of Sleep Disorders (ICSD) [38] was positive. Severe neuroleptic sensitivity was diagnosed when a clinical history was established for both usage of neuroleptic as well as an obvious association of adverse events with the neuroleptic drug. The adverse event was defined as the development or worsening of extrapyramidal features after treatment in the accepted dose range or acute and severe mental or physical deterioration.

Because of the lack of dopamine transporter uptake imaging, the suggestive feature “low dopamine transporter uptake in basal ganglia” on the revised consensus criteria was not applicable in this study. This may have resulted in a lower diagnostic rate for probable DLB.

### Diagnosis of depression

Rates of depression were determined according to the DSM-IV major depression. Diagnosis of current major depressive disorder (MDD) and minor depressive disorder was established using a standardized structured diagnostic interview according to the Mini-International Neuropsychiatric Interview (MINI) 5.0.0 edition [39]. Diagnosis of depression according to the NIMH-dAD provisional criteria was established using a structured diagnostic interview [25]. Severity of depression was determined according to the 17-Item HDRS, the CSDD, and the NPI depression subscale. We also compared each depression symptom in DSM-IV major depression criteria between DLB and AD groups. All patients and their main caregivers were interviewed by a neurologist or a geriatric psychiatrist for the assessment of depressive symptoms. Determination of depression was made by the same consensus meeting composed of three neurologists, one geriatric psychiatrist, and one neuropsychologist.

### Statistics

The Chinese version of SPSS 19.0 for Windows (IBM, SPSS Inc., Chicago) was used for statistical analyses. Comparisons between DLB and AD groups on demographic data, neuropsychological tests, total score of MFCS, motor score of UPDRS, total score of HDRS, total score of CSDD, depression subscale (frequency x severity) of NPI, and the composite scores of NPI were analyzed using independent t-test. Gender and CDR were analyzed with the chi-square test. Two models of multivariable risk estimates for depressive patients compared to non-depressive patients according to the DSM IV major depression criteria were used. In model 1, multivariable odds ratios (ORs) were adjusted for age, education, gender, disease severity, and diagnosis. In model 2, multivariable ORs were adjusted for age, education, gender, disease severity, diagnosis, and antidepressants (Selective serotonin re-uptake inhibitors, Serotonin-norepinephrine reuptake inhibitors, Tricyclic antidepressants, mirtazapine, bupropion, and trazodone). The comparison of frequencies of depressive symptoms between AD and DLB groups was also analyzed with the chi-square test. Crude ORs were adjusted for age and gender and two models of multiple logistic regressions were used in comparing the rate of each depressive symptom in the DLB and AD groups. Model 1 multivariable ORs are derived from a stepwise procedure that considered age, education, gender, disease severity, and depressive symptoms. Model 2 multivariable ORs are derived from a stepwise procedure that considered age, education, gender, disease severity, depressive symptoms, and antidepressants.

### Ethical consideration

The Committee for Medical Research Ethics of Lin-Shin Hospital reviewed the project, and the Data Inspectorate approved it. All participants signed the informed consent when they agreed to join the study.

## Results

A total of 312 patients participated in this study. Among them, 241 patients had AD and 71 patients had DLB. Table 1 demonstrates the demographics and background characteristics of individuals with AD and DLB. Comparison of the demographical information showed significant differences in age, gender, and disease severity. The DLB group had an older age (t = 2.874, df = 310, p = 0.005), a higher male predominance (χ^2^ = 8.025, *df* = 1, p = 0.005) and higher CDR stages (χ^2^ = 15.662, *df* = 2, p < 0.001) than were found in the AD group. Comparisons of the MFCS (t = 4.104, df = 310, p < 0.001), the UPDRS motor subscore (t = 6.897, df = 310, p < 0.001), and the NPI total score (t = 4.159, df = 310, p < 0.001) proved to be significantly more severe for the DLB group than for the AD group. Comparisons of clinical features showed higher frequencies of fluctuation (χ2 = 17.900, df = 1, p < 0.001), VHs (χ2 = 80.328, df = 1, p < 0.001), parkinsonism (χ2 = 81.062, df = 1, p < 0.001), RBD (χ2 = 14.854, df = 1, p < 0.001), and severe neuroleptic sensitivity (χ2 = 12.434, df = 1, p < 0.001) in the DLB group than in the AD group.

**Table 1:** Demographics and background characteristics of individuals with DLB and AD.

	DLB (N = 71)	AD (N = 241)	t/χ^2^	p-value
Age, year (SD, range)	79.7 (6.3, 62–91)	77.1 (8.4, 45–95)	2.874	0.005
Gender, male/female	38/33	84/157	8.025	0.005
Education, year (SD, range)	5.8±5.1 (0–19)	5.4±4.7 (0–16)	0.556	NS
Disease duration, year (SD, range)	2.9±2.1 (1.1–11.0)	3.0±2.3 (1.0–11.0)	-0.378	NS
CDR 0.5/1/2-3	9/30/32	74/110/57	15.662	<0.001
CDR-SB (SD, range)	8.7±3.2 (2.0–15.0)	6.6±3.4 (2.0–17.0)	4.618	<0.001
MMSE (SD, range)	14.9±7.7 (0–28)	17.4±7.0 (0–28)	-2.527	0.012
CASI (SD, range)	48.2±24.4 (0–90)	55.7±23.0 (0–91)	-2.406	0.017
MFCS (SD, range)	1.9±1.2 (0–4)	1.2±1.3 (0–4)	4.104	<0.001
UPDRS-M (SD, range)	17.9±9.8 (1–44)	8.9±8.5 (0–39)	6.897	<0.001
NPI (SD, range)	27.3±15.3 (2–81)	19.0±14.5 (0–68)	4.159	<0.001
Antidepressants*, n (%)	17 (23.9)	55 (22.9)	0.032	NS
Clinical features				
Fluctuation, n (%)	29 (40.8)	41 (17.0)	17.900	<0.001
VHs, n (%)	34 (47.9)	12 (5.0)	80.328	<0.001
Parkinsonism, n (%)	56 (78.9)	51 (21.2)	81.062	<0.001
RBD, n (%)	24 (33.8)	33 (13.7)	14.854	<0.001
Neuroleptic sensitivity, n (%)	9 (12.7)	6 (2.5)	12.434	0.002

N: Number of cases; SD: Standard deviation; NS: Non-significance; AD: Alzheimer’s disease; DLB: Dementia with Lewy bodiese; CDR: Clinical Dementia Rating scale; CDR-SB: Sum of boxes of CDR; MMSE: Mini Mental State Examination; CASI: Cognitive Abilities Screening Instrument; MFCS: Mayo Fluctuation Composite Score; UPDRS-M: Motor subscore of Unified Parkinson’s Disease Rating Scale; NPI: Neuropsychiatric Inventory total score; VHs: Visual hallucinations; RBD: REM sleep behavior disorder;

* Antidepressants include selective serotonin re-uptake inhibitors, serotonin-norepinephrine reuptake inhibitors, tricyclic antidepressants, mirtazapine, bupropion, and trazodone.

The DLB group had a higher frequency of depression according to DSM-IV major depression criteria (χ2 = 5.740, df = 1, p = 0.017) than that of the AD group. Using the criteria, 14 (19.7%) DLB patients and 21 (8.7%) AD patients were diagnosed with major depression. However, the frequency of minor depression (χ2 = 0.441, df = 1, p = 0.506) was not higher in DLB group (31.6%) than that of the AD group (28.2%). Although there was a trend, the frequency of depression according to the NIMH-dAD criteria (χ2 = 2.622, df = 1, p = 0.105) was also not higher in DLB group (43.7%) than that of the AD group (33.2%). Table 2 reveals two models of multivariable risk estimates for depressive patients compared to non-depressive patients according to the DSM IV major depression criteria. In model 1, the results showed that only diagnosis was significantly different (DLB> AD; OR 2.61; 95% CI, 1.19–5.73). In model 2, the results revealed that there were significant differences for only diagnosis (DLB> AD; OR 2.46; 95% CI, 1.08–5.56) and antidepressants (OR 4.40; 95% CI, 1.96–9.90).

**Table 2:** Multivariable risk estimates (odd ratios) for depressive patients compared to non-depressive patients according to the DSM IV major depression criteria.

	N (%)		Model 1			Model 2	
	Depressive	Non-depressive	OR (95%CI)		p-value	OR (95%CI)	p-value
Diagnosis							
DLB	14 (19.7)	57 (80.3)	**2.61 (1.19–5.73)**		**0.017**	**2.46 (1.08–5.56)**	**0.031**
AD	21 (8.7)	220 (91.3)	1			1	
Age, year (SD)	77.6 (8.5)	77.7 (8.1)	1.01 (0.96–1.06)		0.771	0.99 (0.94–1.05)	0.824
Education, year (SD)	5.8 (5.0)	5.8 (4.9)	0.94 (0.87–1.13)		0.169	0.96 (0.88–1.05)	0.349
Gender							
Male	13 (9.0)	131 (90.0)	0.47 (0.19–1.14)		0.094	0.65 (0.26–1.59)	0.341
Female	28 (13.1)	186 (86.9)	1			1	
Disease severity							
CDR≧ 2	17 (16.0)	89 (84.0)	1.92 (0.63–5.85)		0.254	2.28 (0.69–7.52)	0.176
CDR = 1	16 (9.7)	149 (90.3)	1.10 (0.39–3.06)		0.860	0.99 (0.34–2.86)	0.978
CDR = 0.5	8 (9.2)	79 (90.8)	1			1	
Antidepressants*							
Yes	22 (7.9)	256 (92.1)				**4.40 (1.96–9.90)**	**<0.001**
No	19 (24.1)	60 (75.9)					1

Model 1: Multivariable odds ratios are adjusted for age, education, gender, disease severity, and diagnosis. Model 2: Multivariable odds ratios are adjusted for age, education, gender, disease severity, diagnosis, and antidepressants. N: Number of cases; probable DLB: probable Dementia with Lewy bodies; possible DLB: possible Dementia with Lewy bodies; AD: Alzheimer’s disease;

* Antidepressants include selective serotonin re-uptake inhibitors, serotonin-norepinephrine reuptake inhibitors, tricyclic antidepressants, mirtazapine, bupropion, and trazodone.

To avoid the confounding of apathy in the presentation of depression, we further add the apathy domain in NPI into the analysis using multiple logistic regression in both models between two groups. The results did show significantly higher association of apathy in depressed group in model 1 (p = 0.003) and model 2 (p = 0.004); however, the other factors that associated to depressed group are still the same. In model 1, the results showed that only diagnosis was significantly different (DLB> AD; OR 2.46; 95% CI, 1.09–5.54). In model 2, the results revealed that there were significant differences for only diagnosis (DLB> AD; OR 2.43; 95% CI, 1.05–5.46) and antidepressants (OR 4.44; 95% CI, 1.92–10.26).

**Fig 1:**
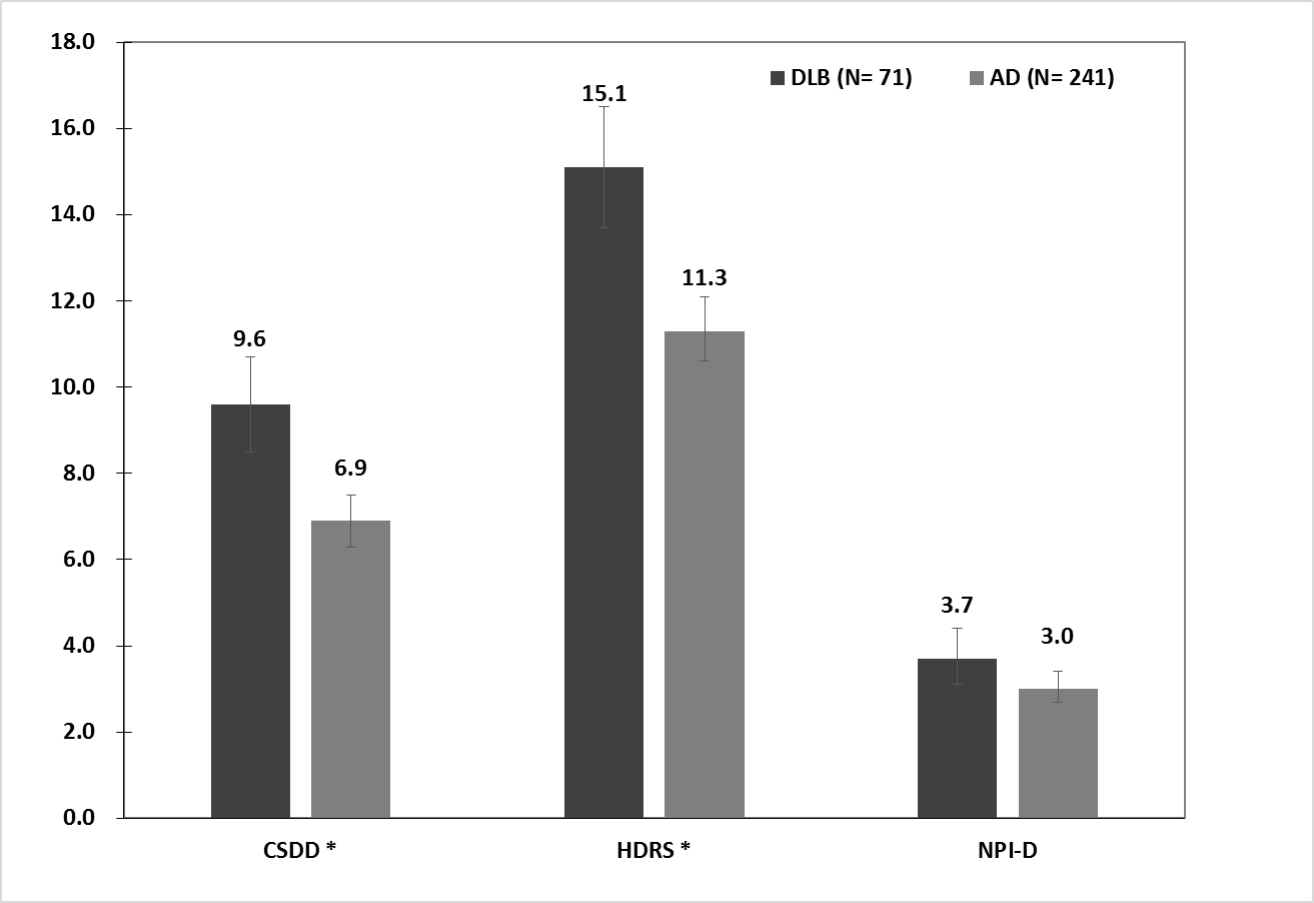
Severity of depression determined with different assessment tools in AD and DLB participants. AD: Alzheimer’s disease; DLB: Dementia with Lewy bodies; CSDD: Total score of Cornell Scale for Depression in Dementia; HDRS: Total score of Hamilton Depression Rating Scale; NPI-D: Depression subscore of neuropsychiatric inventory (NPI). * p < 0.005. Error bars represent 95% confidence intervals.

Fig 1 illustrates the severity of depression as determined with different assessment tools in the AD and DLB participants. The severity of depression according to the 17-Item HDRS and the CSDD was significantly different. Higher HDRS (t = 4.659, df = 310, p < 0.001) and CSDD (t = 4.375, df = 310, p < 0.001) in the DLB group than in the AD group. The NPI depression subscale was not different between the two groups (t = 1.721, df = 310, p = 0.086).

**Fig 2:**
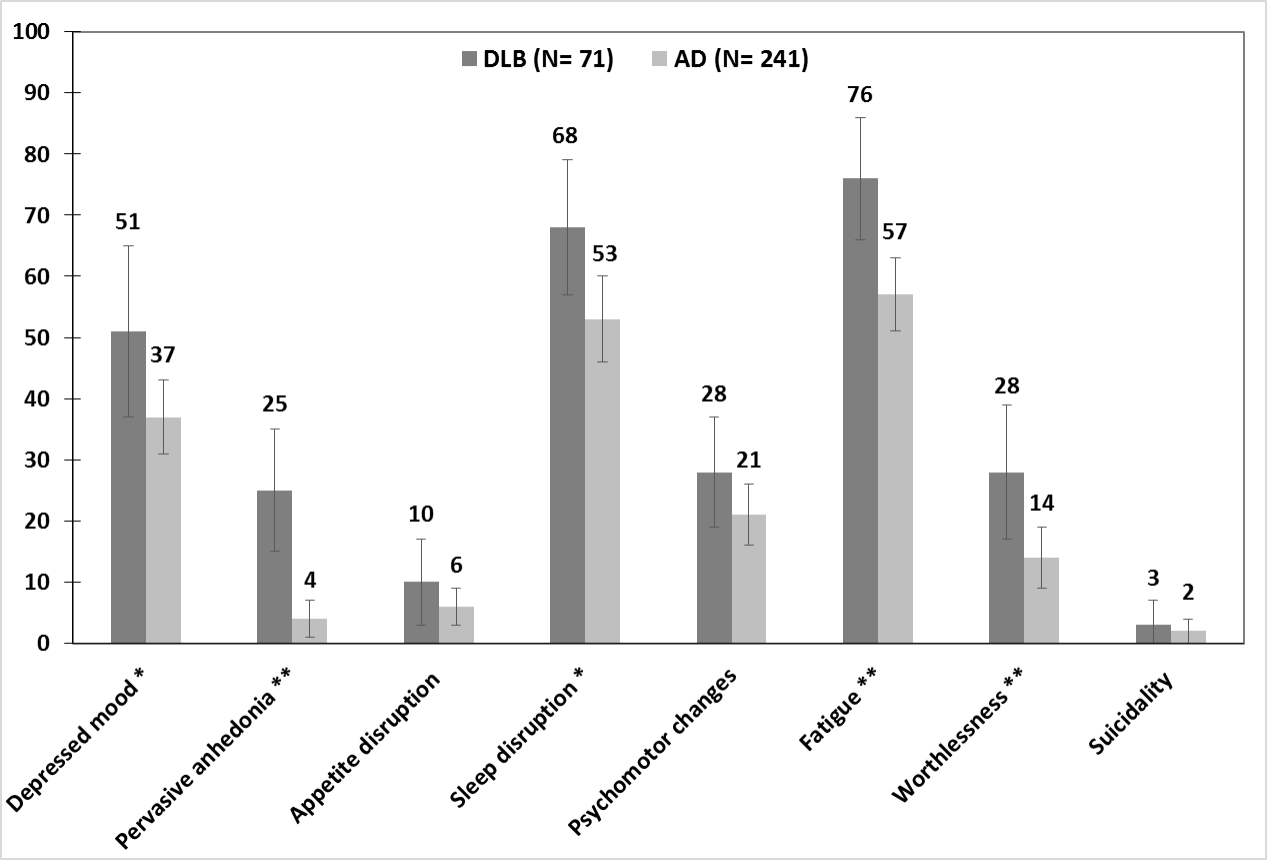
Frequencies of individual depressive symptoms in AD, probable DLB, possible DLB, and DLB participants. AD: Alzheimer’s disease; probable DLB: probable Dementia with Lewy bodies; possible DLB: possible Dementia with Lewy bodies; DLB: probable and possible Dementia with Lewy bodies. * p < 0.05; ** p < 0.005. Error bars represent 95% confidence intervals.

The frequency of depressive symptoms according to DSM-IV major depression criteria between the two groups is shown in Fig 2.

Table 3 lists the crude and two models of multivariable risk estimates for depressive symptoms for the DLB and AD groups. Crude ORs were higher in the DLB group in the symptoms of persistent depressed mood (OR 1.76; 95% CI, 1.03 to 3.00), pervasive anhedonia (OR 8.76; 95% CI, 3.73 to 20.57), sleep disruption (OR 1.87; 95% CI, 1.07 to 3.27), fatigue (OR 2.37; 95% CI, 1.30 to 4.33), and worthlessness (OR 2.47; 95% CI, 1.31 to 4.66). Model 1 multivariable ORs were higher in DLB in the symptoms of pervasive anhedonia (OR 6.97; 95% CI, 2.70 to 18.01), sleep disruption (OR 1.95; 95% CI, 1.03 to 3.69), and worthlessness (OR 2.17; 95% CI, 1.06 to 4.44). We further adjusted antidepressants used in model 2, with the result demonstrating the same result of pervasive anhedonia (OR 6.87; 95% CI, 2.46 to 17.84), sleep disruption (OR 1.92; 95% CI, 1.01 to 3.66), and worthlessness (OR 2.14; 95% CI, 1.04 to 4.41). being significantly higher in the DLB group than in the AD group. Among the depressive symptoms, pervasive anhedonia had the highest ORs (crude/model 1/model 2 = 8.76/6.97/6.87) for the diagnosis of DLB compared to that of AD.

**Table 3:** Crude and multivariable risk estimates (odd ratios) for depressive symptoms between DLB and AD.

Depressive symptoms	Crude**		Model 1***			Model 2****		
	OR (95% CI)	p-value	OR (95%CI)		p-value	OR (95%CI)		p-value
Depressed mood	**1.76 (1.03–3.00)**	**0.037**	*			*		
Pervasive anhedonia	**8.76 (3.73–20.57)**	**<0.001**	**6.97 (2.70–18.01)**		**<0.001**	**6.87 (2.64–17.84)**		**<0.001**
Appetite disruption	1.65 (0.64–4.22)	0.293	*			*		
Sleep disruption	**1.87 (1.07–3.27)**	**0.026**	**1.95 (1.03–3.69)**		**0.040**	**1.92 (1.01–3.66)**		**0.047**
Psychomotor changes	1.50 (0.64–4.22)	5.8 (4.9)	*			*		
Fatigue	**2.37 (1.30–4.33)**	**0.004**	*			*		
Worthlessness	**2.47 (1.31–4.66)**	**0.004**	**2.17 (1.06–4.44)**		**0.034**	**2.14 (1.04–4.41)**		**0.038**
Difficulty concentrating	1.30 (0.64–4.22)	0.356	*			*		
Suicidality	1.37 (0.26–7.21)	0.660	*			*		

AD: Alzheimer’s disease; DLB: Dementia with Lewy bodies; probable DLB: probable Dementia with Lewy bodies; possible DLB: possible Dementia with Lewy bodies. The odds ratio (OR) and 95% confidence interval (CI) were calculated with the AD group as reference.

*ORs absent for variables not included in multivariable regression analysis.

**Crude ORs are adjusted for age and gender;

***Model 1 multivariable ORs are derived from a stepwise procedure that considered age, education, gender, disease severity, and all depressive symptoms;

****Model 2, multivariable ORs are derived from a stepwise procedure that considered age, education, gender, disease severity, antidepressants, and all depressivesymptoms.

## Discussion

This study has provided data directly focusing on the association of depression and depressive symptoms with DLB and AD by using DSM-IV criteria, NIMH-dAD provisional criteria, and multiple rating scales. This study has also demonstrated the frequency and severity of depression, as well as the comparison of depressive symptoms, between DLB and AD groups.

The first part of this study deals with the frequency of depression in DLB and AD, and found that co-morbid depression was more frequent in DLB (19.7%) compared with that for AD (8.7%) using the DSM-IV major depression criteria. This finding is consistent with most of the previous studies that compared DLB with AD or other types of neurodegenerative disorders and found that DLB showed higher rates of depression. For example, a review of four earlier studies found that regardless of disease stages, major depression was significantly more common in DLB (24.45%) than in AD (9.32%) [40]. The rate of depression associated with DLB according to the DSM-IV major depression criteria was also found to be similar to that of a study of psychiatric symptoms in DLB (19.4%) [41]. In our study, the NIMH-dAD criteria is first used for the diagnosis of depression in DLB and the rate of depression in DLB is not significantly higher in DLB (43.7%) than AD (33.2%). This finding does not meet our original hypothesis that frequency in DLB will be higher when determined by NIMH-dAD criteria. Our results also demonstrate that rates of depression determined with NIMH-dAD criteria in both AD and DLB were higher than those with DSM-IV criteria for major depression. Why did DLB not demonstrate a higher frequency of depression compared with AD when using the NIMH-dAD criteria? First, we propose that this most likely occurs because the NIMH criteria are more inclusive than the DSM-IV in which depression symptoms must be present for most of the day and nearly every day for at least two weeks in order to be scored positive with major depression criteria. However, symptoms that have been present during the same 2-week period and represent a change from previous functioning can be scored positive with NIMH-dAD criteria. Therefore, the NIMH criteria may minimize the risk of excluding patients with AD who suffer from clinically significant affective disturbances [26, 28]. Second, although the rate of depression according to the NIMH-dAD criteria is not significantly higher in DLB than in AD, there is still a trend (43.7%: 33.2%; χ2 = 2.622, df = 1, p = 0.105) that implicated a higher rate depression in DLB. We proposed that if more participants were enrolled, the result might reach a significant difference.

The second part of this study focused on the severity of depression in the DLB and AD groups, and our results consistently revealed that more severe depression was found in probable DLB than in AD using multiple rating scales, including the CSDD and HDRS. However, the NPI depression subscale did not demonstrate a significant difference among the three groups. Findings from previous studies that compared the severity of depression between AD and DLB are equivocal. A recent study of the Japanese population using the GDS (Geriatric depression scale) showed a higher total score for severity of depression in DLB than in AD (16.1 vs. 7.8 respectively; P = 1.37E-22) [29]. A prospective, longitudinal Belgian study on neuropsychiatric symptoms of dementia using the CSDD to study depression in AD, DLB and other dementias showed no difference in total CSDD scores [24]. In the Belgian study, the difference of total CSDD score between two groups (AD/DLB = 5.9/7.9) is similar to our result (AD/DLB = 6.9/9.6); however, the results of their study did not reach a level of significance probably due to the small number of DLB patients (n = 23). These discrepancies may also be attributable to method differences in assessment. For example, the severity and frequency necessary for the diagnosis of depression in NPI are less restrictive and therefore may include mild depression and occasionally experienced depressive symptoms. Furthermore, as HDRS, NPI and CSDD are based on hetero-evaluation, GDS is an auto-evaluation. Discrepancy between the two types of evaluation occurred among mildly as well as moderately demented patients when insight was impaired [42].

The third part of this study regarded to the difference of depressive symptoms between DLB and AD. Both adjusted models demonstrated the same finding of pervasive anhedonia, sleep disruption, and worthlessness being significantly higher in the DLB than in the AD group. Pervasive anhedonia had the highest ORs (crude/model 1/model 2 = 8.76/6.97/6.87) in DLB compared with AD. Based on these findings, we propose that pervasive anhedonia has the greatest value for the differential diagnosis of depressive symptoms between DLB and AD. Few previous studies have directly compared the depressive symptoms between DLB and AD. The study on autopsy-confirmed DLB and AD conducted by Samuels et al. failed to showed any difference in depressive symptoms between AD and DLB. They also concluded that the presence or absence of depression couldn’t be used to distinguish between AD and DLB [21]. In the study, Samuels et al. analyzed a relatively small sample size of autopsy-confirmed cases of DLB (N = 16) and AD (N = 39). Besides, they used postmortem chart review (PMCR) for clinical assessment of depression. It could be less sensitive and less precise to determine depression with PMCR than with a structured interview according to the Mini-International Neuropsychiatric Interview (MINI) [39]. However, based on the pathophysiological and the neurochemical differences between DLB and AD, we hypothesized that the depressive symptoms would be different between DLB and AD, especially those symptoms associated with dopamine deficits [10,11], and our results successfully confirm this hypothesis.

Finally, although some previous studies have shown that depression is more frequent and more severe in females, this finding may not be necessarily applicable for patients with dementia based on the result of higher and more severe depression among DLB patients, among whom, males were more frequently involved in our study.

The study has limitations that should be noted. First, our research was conducted in only one hospital in central Taiwan. Therefore, selection bias may arise, and our findings may not be generalizable to all patients with DLB or AD. Second, the comparison of depression between DLB and AD in our study was cross-sectional. Therefore, the frequency or severity of depression may change along with the severity of dementia. Third, because of the lack of more determinative equipment or facilities, such as genetic study, dopamine transporter uptake imaging, amyloid plaque imaging, CSF biomarkers, or pathological studies, the diagnosis of DLB and AD was based only on clinical criteria. For this reason, diagnosis bias may arise.

In conclusion, our findings suggest that co-morbid major depression is more frequent in DLB than in AD. Co-morbid depression was more severe in DLB than in AD using HDRS and CSDD among all patients. Among depressive symptoms, pervasive anhedonia had the greatest value for the differential diagnosis of depression between the DLB and AD groups. Besides, this is the first study that using NIMH-dAD criteria for the diagnosis of depression in DLB. Rates of depression determined with NIMH-dAD criteria in both AD and DLB were higher than those with DSM-IV criteria for major depression. Although there was a trend, concurrence of depression according to the NIMH-dAD criteria was not significantly higher in DLB than in AD.
